# Chemical Characterization of *N*-Linked Oligosaccharide As the Antigen Epitope Recognized by an Anti-Sperm Auto-Monoclonal Antibody, Ts4

**DOI:** 10.1371/journal.pone.0133784

**Published:** 2015-07-29

**Authors:** Hiroshi Yoshitake, Noritaka Hashii, Nana Kawasaki, Shuichiro Endo, Kenji Takamori, Akiko Hasegawa, Hiroshi Fujiwara, Yoshihiko Araki

**Affiliations:** 1 Institute for Environmental and Gender-specific Medicine, Juntendo University Graduate School of Medicine, Urayasu, Chiba, Japan; 2 Division of Biological Chemistry and Biologicals, National Institute of Health Sciences, Setagaya-ku, Tokyo, Japan; 3 Department of Obstetrics and Gynecology, Juntendo University Graduate School of Medicine, Bunkyo-ku, Tokyo, Japan; 4 Department of Obstetrics and Gynecology, Hyogo College of Medicine, Nishinomiya, Hyogo, Japan; 5 Department of Obstetrics and Gynecology, Kanazawa University Graduate School of Medical Science, Kanazawa, Ishikawa, Japan; University of the Witwatersrand, SOUTH AFRICA

## Abstract

Ts4, an anti-sperm auto-monoclonal antibody, possesses immunoreactivity to the acrosomal region of mouse epididymal spermatozoa. In addition, the mAb shows specific immunoreactivity to reproduction-related regions such as testicular germ cells and early embryo. Our qualitative study previously showed that the antigen epitope for Ts4 contained a *N*-linked common oligosaccharide (OS) chain on testicular glycoproteins as determined by Western blotting for testicular glycoproteins after treatment with several glycohydrolases. Since the distribution of the Ts4-epitope is unique, the OS chain in Ts4-epitope may have role(s) in the reproductive process. The aim of this study was to clarify the molecular structure of the Ts4-epitope, particularly its OS moiety. Using Ts4 immunoprecipitation combined with liquid chromatography and multiple-stage mass spectrometry, the candidate carbohydrate structure in the Ts4-epitope is proposed to be *N*-linked fucosylated agalacto-biantennary with bisecting *N*-acetylglucosamine (GlcNAc) or with *N*-acetylgalactosamine-GlcNAc motif. Further binding analyses using various lectins against the mouse testicular Ts4-immunoprecipitants revealed that *Phaseolus vulgaris* erythroagglutinin and *Pisum sativum* agglutinin showed positive staining of the bands corresponding to Ts4 reactive proteins. Moreover, the immunoreactivity of Ts4 against the testicular extract was completely abrogated after digestion with β-*N*-acetylglucosaminidase. These results show that the Ts4-epitope contains agalacto-biantennary *N*-glycan with bisecting GlcNAc carrying fucose residues.

## Introduction

To clarify molecular mechanisms on idiopathic infertility, we are currently focusing on anti-sperm auto-antibodies (ASA-Abs), occasionally identified in the sera of infertile males [1]. As an approach to investigate immunological effects of ASA-Abs on mammalian fertilization, we previously generated several ASA-monoclonal Abs (mAbs) using spleen cells from aged mice (> 1 year old) maintained under conventional conditions [2]. Among the mAbs established, we focused on one ASA-mAb, designated Ts4, which exhibited the following characteristics: 1) Specific immunoreactivity was observed against reproduction-related regions, including the acrosomal region of spermatozoa isolated from mouse epididymis, the plasma membranes of spermatocytes, spermatids, and testicular spermatozoa within the seminiferous tubules, and the early embryo, but not in other somatic cells of major organs [2, 3] (summarized in Table 1); 2) Western blotting with this mAb of the epididymal and testicular extracts detected several bands [3]; and 3) Ts4 immunoreactivity vanished upon pretreatment of the testicular extract with *N*-glycanase, an endoglycosidase known to cleave all types (high-mannose/hybrid, bi-, tri-, and tetraantennary complex-type) of *N*-linked oligosaccharide (OS) chains [2]. These results suggest that the Ts4 mAb immunoreacts with several kinds of glycoproteins possessing a unique common OS structure, in the acrosomal region of epididymal spermatozoa and on the plasma membranes of testicular germ cells, via binding to epitopes containing the specific *N*-linked OS chain on the molecules.

**Table 1:** Profiles of expression patterns of Ts4-reactive molecule(s) and TEX101 in male and female germ cells, embryonic cells, and somatic cells by immunomorphological analysis ^a^.

Organ		Expression of Ts4-reactive molecule(s)	Expression of TEX101
Testis			
	Spermatogonia	–	–
	Spermatocyte	+	+
	Round spermatid	+	+
	Elongated spermatid	+	+
	Testicular sperm	+	+
	Interstitial cell ^b^	–	–
	Sertoli cell	–	–
Epididymis			
	*Epididymal sperm	+	–
	Epithelium	–	–
Ovary			
	Oocyte	–	–
	Granulosa cell	–	–
Embryo			
	*4-cell stage	+	–
	*blastocyst	+	–
	(*ES cell)	+	–
Somatic organs (adult)			
	Brain	–	–
	Heart	–	–
	Lung	–	–
	Stomach	–	–
	Liver	–	–
	Spleen	–	–
	Kidney	–	–

^a^ Data from references (2, 3).

^b^ Interstitial cells include leydig cells.

+ Positive,

–Negative.

* Cells that possess Ts4-reactive molecules but not TEX101.

Although identification of the Ts4-reactive glycoproteins on the acrosomal region of epididymal spermatozoa has not yet been clarified, we previously demonstrated that one of the Ts4-recognozed testicular molecules is TEX101, a glycosylphosphatidyl inositol-anchored glycoprotein [2, 4, 5]. This implies that the Ts4-reactive OS chain is, at least, associated with TEX101. Immunofluorescent studies using a mAb for a peptide portion of TEX101 showed that this molecule is a specific marker for both male and female germ cells during gonadal development and the on and off switching of TEX101 expression in germ cells nearly parallels the kinetics of gametogenesis, but it disappears from mature germ cells (spermatozoa in the cauda epididymis and oocytes in the secondary follicles) [2, 5–7]. Interestingly, unlike the distribution of TEX101, the immunoreactivity of Ts4 remains even in the mature germ cells (epididymal spermatozoa), fertilized eggs and early embryos as if it is transferred from TEX101 to unidentified glycoproteins [2, 3] (Table 1). From a reproductive biological point of view, the Ts4-epitope in TEX101 seems to be a specific glycan that is passed from germ cells to fertilized eggs like "a baton pass of the relay", changing the host-protein(s) that presumably necessary to maintain steric structure for biologically activity, if it possesses.

Glycosylation is one of the most pivotal posttranslational modifications and is involved in biological processes, such as cell-cell or cell-extracellular matrix interactions and cell differentiation and proliferation [8]. Based on these findings, we speculated that the specific *N*-glycan in the epitope for Ts4 may contribute to male germ cell differentiation and proliferation, fertilization, and embryonic development via modulating functions of the Ts4-reactive *N*-glycan-conjugated glycoproteins. As an initial analysis of the *N*-glycan in the Ts4-epitope, we determined the structure of *N*-glycans that formed the Ts4-reactive epitope of TEX101 within the mouse testis using an immunoprecipitation method combined with liquid chromatography and multiple-stage mass spectrometry (LC/MS) in the present report.

## Materials and Methods

### Animals

Male ICR mice (6-12-week-old) were purchased from Sankyo Lab Service (Tokyo, Japan). They were maintained at the Animal facilities of Juntendo University under 12L:12D conditions, and given free access to food and water. All animal experiments were conducted according to the guide for care and use of laboratory animals, Juntendo University.

### Antibodies and reagents

The Ts4 mAb (mouse IgM) and 6035 (a mAb for TEX101 peptide portion (mouse IgG2a)) were established and purified as previously described [2]. Horseradish peroxidase (HRP)-conjugated rabbit anti-mouse IgG polyclonal antibody (pAb) and control mouse IgM were obtained from DAKO (Glostrup, Denmark). HRP-conjugated goat anti-mouse IgM pAb were purchased from Chemicon (Temecula, CA). Various biotin-labeled lectins, such as *Dolichos biflorus* agglutinin (DBA), *Datura stramonium* agglutinin (DSA), *Phaseolus vulgaris* erythroagglutinin (PHA-E), *Phaseolus vulgaris* leucoagglutinin (PHA-L), *Pisum sativum* agglutinin (PSA), *Sophora japonica* agglutinin (SJA), *Triticum vulgaris* (wheat germ) agglutinin (WGA) were purchased from Vector Laboratories (Burlingame, CA). HRP-conjugated streptavidin was obtained from Invitrogen (Carlsbad, CA). Endoglycosidases (*N*-glycanase, endoglycosidase-F1, endoglycosidase-F2, endoglycosidase-F3, and endoglycosidase-H) and exoglycosidases (α-mannosidase, β-mannosidase, β-*N*-acetylglucosaminidase (from *Canavalia ensiformis*), neuraminidase, and α-L-fucosidase) were from Sigma-Aldrich (St. Louis, MO). β-*N*-acetylglucosaminidase (from *Xanthomonas manihotis*) was purchased from New England Biolabs (Ipswich, MA). Other chemicals used in this study were obtained commercially and were of the highest purity available.

### Preparation of mouse testicular extract and Western blot analysis

The mouse testicular Triton X-100-soluble (TS) extracts were prepared as previously described [2]. Briefly, mature male mice were anesthetized with 3% (v/v) sevoflurane (Pfizer Japan Inc., Tokyo, Japan) in oxygen. Prior to testes isolation, total blood was eliminated by perfusion from left ventricle with phosphate buffered saline (PBS; pH 7.4) under anesthesia. An isolated testis was homogenized with a glass homogenizer 20 strokes in 1 ml of PBS (pH 7.4) containing 1 × EDTA-free Complete inhibitor cocktail (Roche Diagnostics, Penzberg, Germany) (buffer A). After the centrifugation at 18,000 × g for 15 min, the precipitate was resuspended in 1 ml of the buffer A containing 1% Triton X-100 and then extracted by a Polytron PT3100 homogenizer (Kinematica AG, Littau-Lucerme, Switzerland) for 10 sec. Following a 20-min incubation on ice, the suspension was centrifuged at 18,000 × g for 15 min, and the resultant supernatant was used in the following experiments as the testicular TS extract.

The testicular TS extract was separated by SDS-PAGE system [9]. The protein components resolved by SDS-PAGE were electrophoretically blotted onto a polyvinylidene fluoride (PVDF) membrane (Millipore, Bedford, MA), and then the reactivity of the transferred proteins with primary Abs (0.5–1 μg/ml) or several types of biotin-labeled lectins was assayed using HRP-conjugated secondary antibodies or streptavidin and an ECL Western blotting detection system (GE Healthcare, Buckinghamshire, UK) [4, 10].

### Immunoprecipitation with Ts4

Immunoprecipitation was performed as described elsewhere [7]. In brief, prior to the immunoprecipitation, Ts4 or control mouse IgM (each 80 μg) was conjugated with 2 mg of Dynabeads MyOne Tosylactivated (Invitrogen) according to manufacturer’s instructions. Total proteins (50 μg) of the TS testicular extract in buffer A containing 0.1% Triton X-100 was treated with the Ts4 or normal control IgM-conjugated Dynabeads on a rotary shaker at 4°C overnight. After washing with PBS three times, the beads were boiled for 5 min in 3 × SDS buffer [9] under reducing conditions. After the centrifugation, the supernatants were used as samples for SDS-PAGE.

### Release of *N*-linked OS chains by in-gel Peptide-*N*-Glycosidase (PNGase) F digestion

After immunoprecipitation with Ts4, the precipitants were separated by SDS-PAGE, then the gel was stained with coomassie brilliant blue (CBB) using EZBlue Gel Staining Reagent (Sigma-Aldrich) according to manufacturer’s instructions. To isolate *N*-linked OS chains from the gel, in-gel PNGase F digestion was performed as previously described [11]. Briefly, the protein bands were excised from the CBB-stained gel, cut into small pieces, and de-stained with 30% acetonitrile. The de-stained gel pieces were dehydrated with 50% acetonitrile. The dried gels were immersed in reducing agent solution containing 10 mM dithiothreitol and 25 mM ammonium bicarbonate, and then incubated for 60 min at 56°C. After reduction, the gels were incubated with shaking in an alkylation agent solution containing 55 mM sodium monoiodoacetate and 25 mM ammonium bicarbonate for 45 min at room temperature. After washing with 25 mM ammonium bicarbonate and ultrapure water, the gels were dehydrated with 50% acetonitrile. The dried gels were then incubated in 50 mM sodium phosphate buffer (pH 7.2) containing 5 U of PNGase F (Roche Diagnostics) for 16 h at 37°C. The released *N*-glycans were extracted 3 times from the gel pieces by intermittent sonication for 30 min in ultrapure water. All extracts were combined and lyophilized. The released *N*-glycans were reduced with 0.5 M sodium borohydride for 2 h at room temperature. After neutralization with 5% acetic acid, the reduced *N*-glycans were desalted with a solid-phase extraction cartridge (EnviCarb C; Supelco, Bellefonte, PA), lyophilized, and re-dissolved in 25 μl of ultrapure water.

### LC/MS analysis

The reduced *N*-glycans prepared as described above were separated on a graphitized carbon column (Hypercarb, 150 × 0.075 mm, 5 μm; Thermo Fisher Scientific, Waltham, MA) at a flow rate of 300 nl/min in an UltiMate 3000 RSLCnano LC system (Dionex, Sunnyvale, CA). The mobile phases were 5 mM ammonium bicarbonate containing 2% acetonitrile and 5 mM ammonium bicarbonate containing 80% acetonitrile. The glycans were eluted with a linear gradient of 5–60% of 5 mM ammonium bicarbonate containing 80% acetonitrile for 60 min. Structural analysis was performed using a Fourier transform ion cyclotron resonance/ion trap type mass spectrometry (FT-ICR-MS) (LTQ-FT; Thermo Fisher Scientific). For MS, the electrospray voltage was 2.5 kV in both the positive and negative ion modes. The capillary temperature was 200°C, and the collision energy was 25% for the multiple-stage MS (MS^n^) experiment. The resolution of FT-ICR-MS was 50,000, and the scan range was *m/z* 700–2,000. The sample solution of 5 μl was injected into the LC/MS.

## Results

### Confirmation of Ts4-immunoreactivity against OS moiety

**Fig 1:**
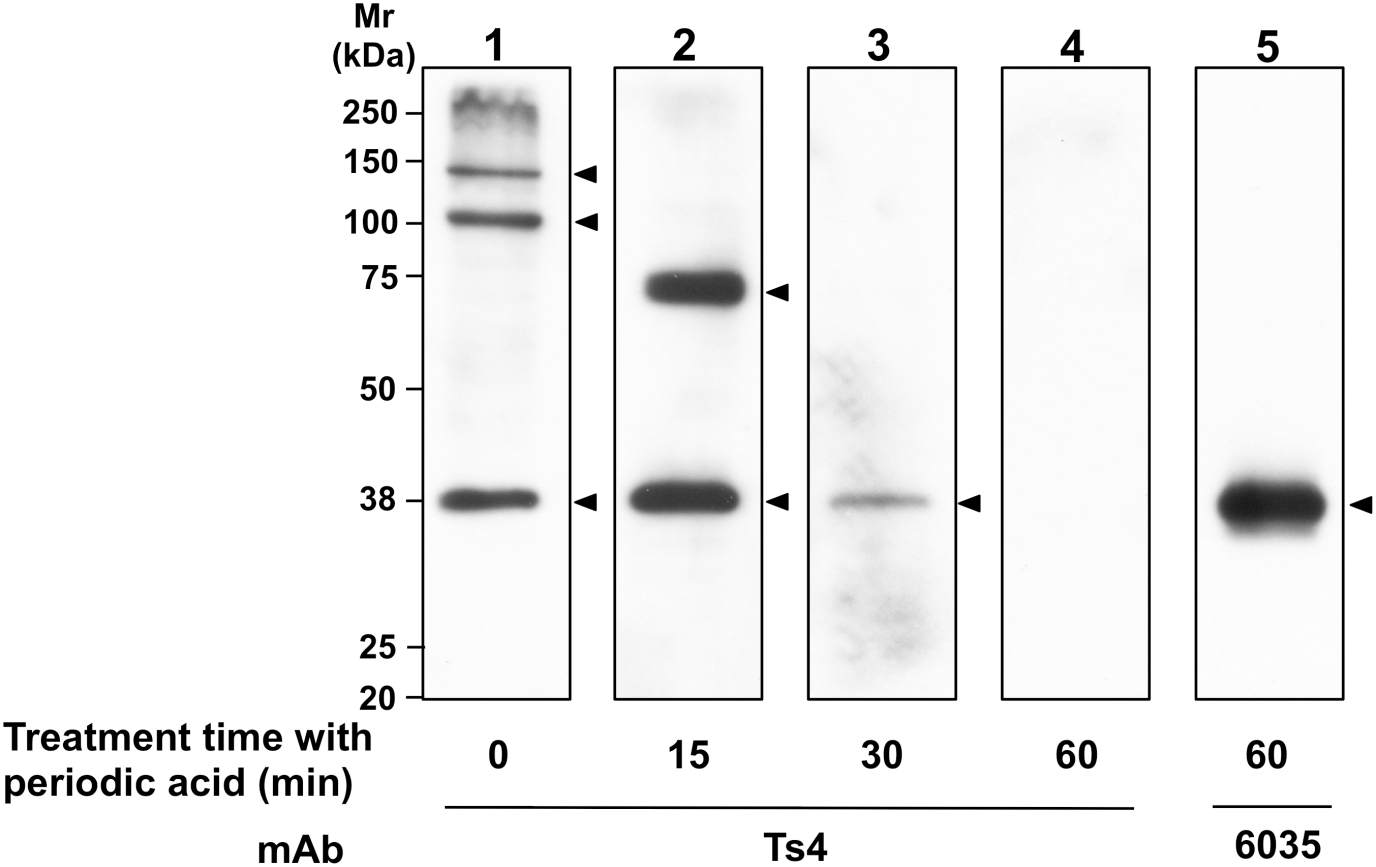
Immunoreactivity of Ts4 against testicular proteins pretreated with periodic acid. The testicular TS fractions (each 5 μg protein) were loaded on a 10% gel, separated by SDS-PAGE under reducing conditions, and then blotted onto a PVDF membrane. The PVDF membrane was divided into individual lanes, which were treated with 0.075 M NaIO_4_ and HIO_4_ 2H_2_O in PBS for various times. Specific bands were detected with Ts4 or 6035 (arrowheads). Mr, molecular mass.

Before structural analysis, we examined whether all epitopes for Ts4 in testicular Triton X-100-soluble (TS) extracts contain OS chains. Western blot analyses revealed that Ts4 detected approximately 38-, 110-, and 140-kDa bands in the testicular extract (Fig 1, lane 1) confirming previously described experimental result [2]. After periodic acid treatment which linearizes the hexose ring and destroys OS chains [12, 13], all Ts4 reactive bands of the testicular TS extract electrotransferred on PVDF membranes lost their immunogenicity in a time-dependent manner (Fig 1, lanes 2–4). Bands of apparent molecular masses at 110- and 140-kDa were completely abrogated after 15 min treatment with periodic acid, whereas the bands at 38-kDa were traced after 30-min of treatment (Fig 1, lanes 2, 3). We previously reported that the 38-kDa band detected with Ts4 corresponds to TEX101, and that the molecular epitope on TEX101 for Ts4 contains *N*-linked OS chain [2]. Although the peptide moiety of the 38-kDa band (TEX101) can be detected by 6035 (a specific mAb against the TEX101-peptide portion [2]) even after treatment with periodic acid, the Ts4 reactive bands were entirely disappeared within 60 min after the treatment (Fig 1, lanes 4, 5). Taken together, these results clearly show that nature of the all antigen epitopes on testicular proteins for Ts4 is carbohydrate, presumably *N*-linked OS chain.

### OS profiling by LC/MS

**Fig 2:**
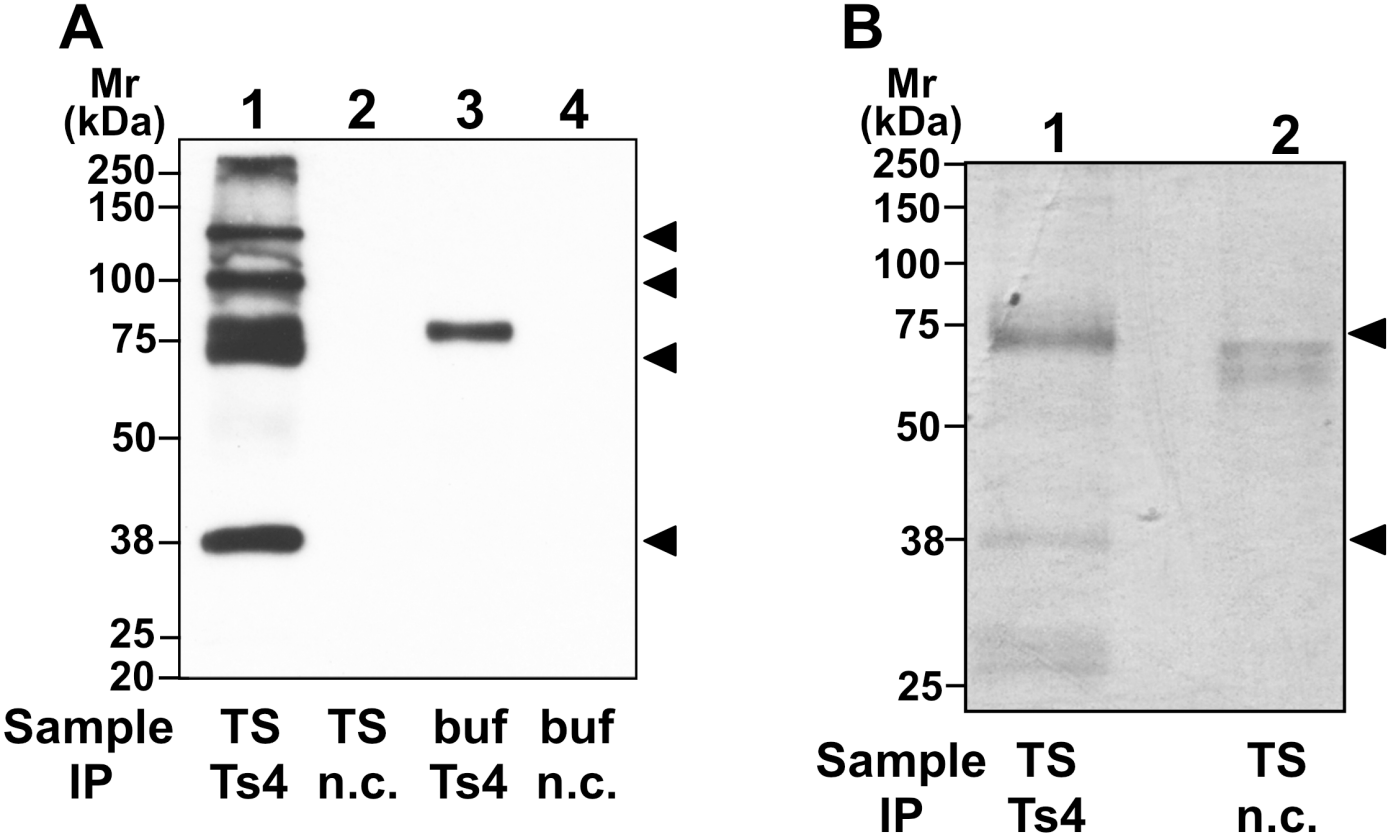
SDS-PAGE analyses of mouse testicular proteins immunoprecipitated with Ts4. Western blot analyses using Ts4 (A). Testicular TS proteins immunoprecipitated with either Ts4 or normal control mouse IgM (n.c.) were separated via SDS-PAGE on 10% gels under reducing conditions. Control experiments were conducted under the same conditions, but in the absence of the testicular extract (buf). Separated proteins were electroblotted onto PVDF membranes and then probed with Ts4. Arrowheads indicate molecular mass (Mr) of the specific immunoreactive bands. Visualized by CBB-staining (B). The same samples were applied to lanes of the 10% SDS-PAGE gel under reducing conditions, and then the gel was CBB-stained. Apparent positions of dominant bands obtained via immunoprecipitation with Ts4 are indicated with arrowheads.

To identify the *N*-glycan contents recognized by Ts4, we adopted a glycomic approach. We initially isolated proteins immunoreacted with Ts4 from the testicular TS extract using immunoprecipitation. Western blot analyses of the Ts4-immunoprecipitants showed bands at apparent molecular masses of 140-, 110-, 70- 38-kDa detected with the same mAb (Fig 2A, lane 1). The adsorbed proteins in the same immunoprecipitants were separated by SDS-PAGE, and then the gel was visualized by CBB staining. Two specific bands at approximately 38 and 70 kDa were observed in the Ts4-precipitated proteins (Fig 2B, lane 1).

**Fig 3:**
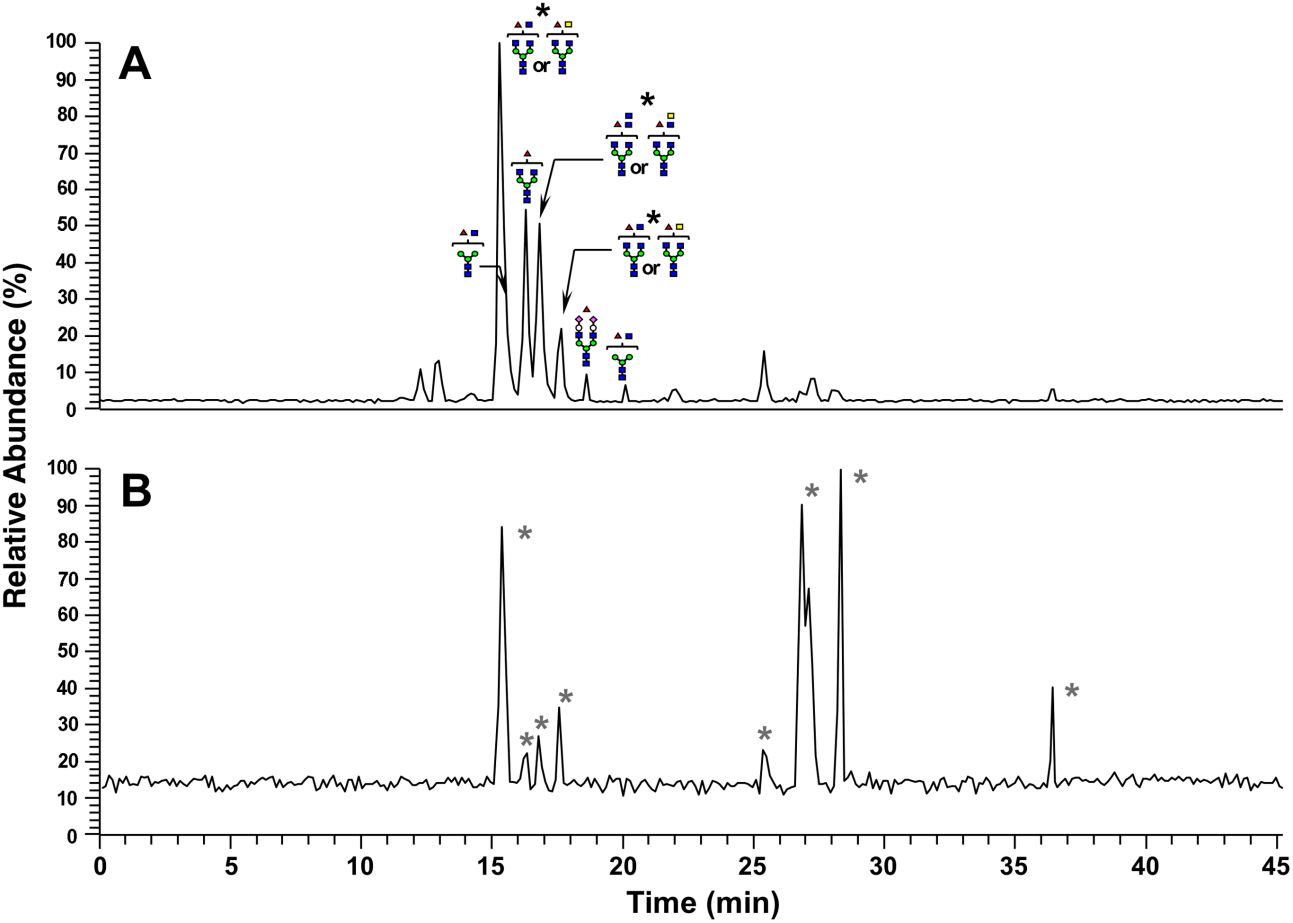
The total ion current chromatogram of *N*-glycans from the 38-kDa band obtained by FT-ICR-MS (*m/z* 700–2,000). The positive-ion mode (A) and the negative-ion mode (B). Green circle: mannose, white circle: galactose, red triangle: fucose, blue square: GlcNAc, yellow square: GalNAc, pink diamond: *N*-acetylneuraminic acid (NeuNAc), black asterisk: glycan carrying bisecting GlcNAc or LacdiNAc, gray asterisk: non-glycan peak.

**Fig 4:**
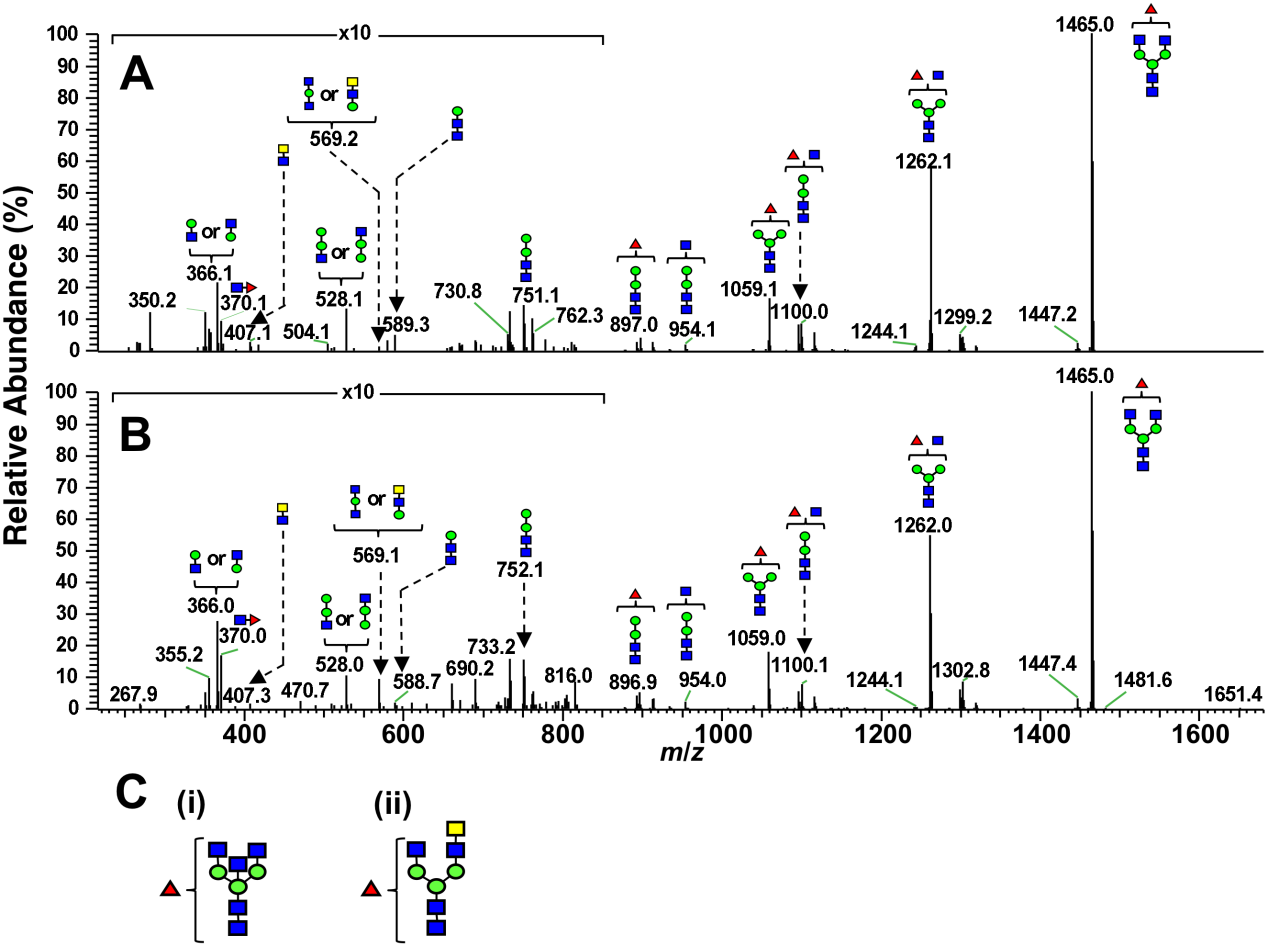
The MS^2^ spectra of main glycans [dHex1Hex3HexNAc5 (1) in S1A and S1B Fig]. The 38-kDa band (A) and 70-kDa band (B). The potential deduced structures of *N*-glycan as the Ts4-epitope (C). Bisecting GlcNAc (i), LacdiNAc (ii). Green circle: mannose, red triangle: fucose, blue square: GlcNAc, yellow square: GalNAc.

**Fig 5:**
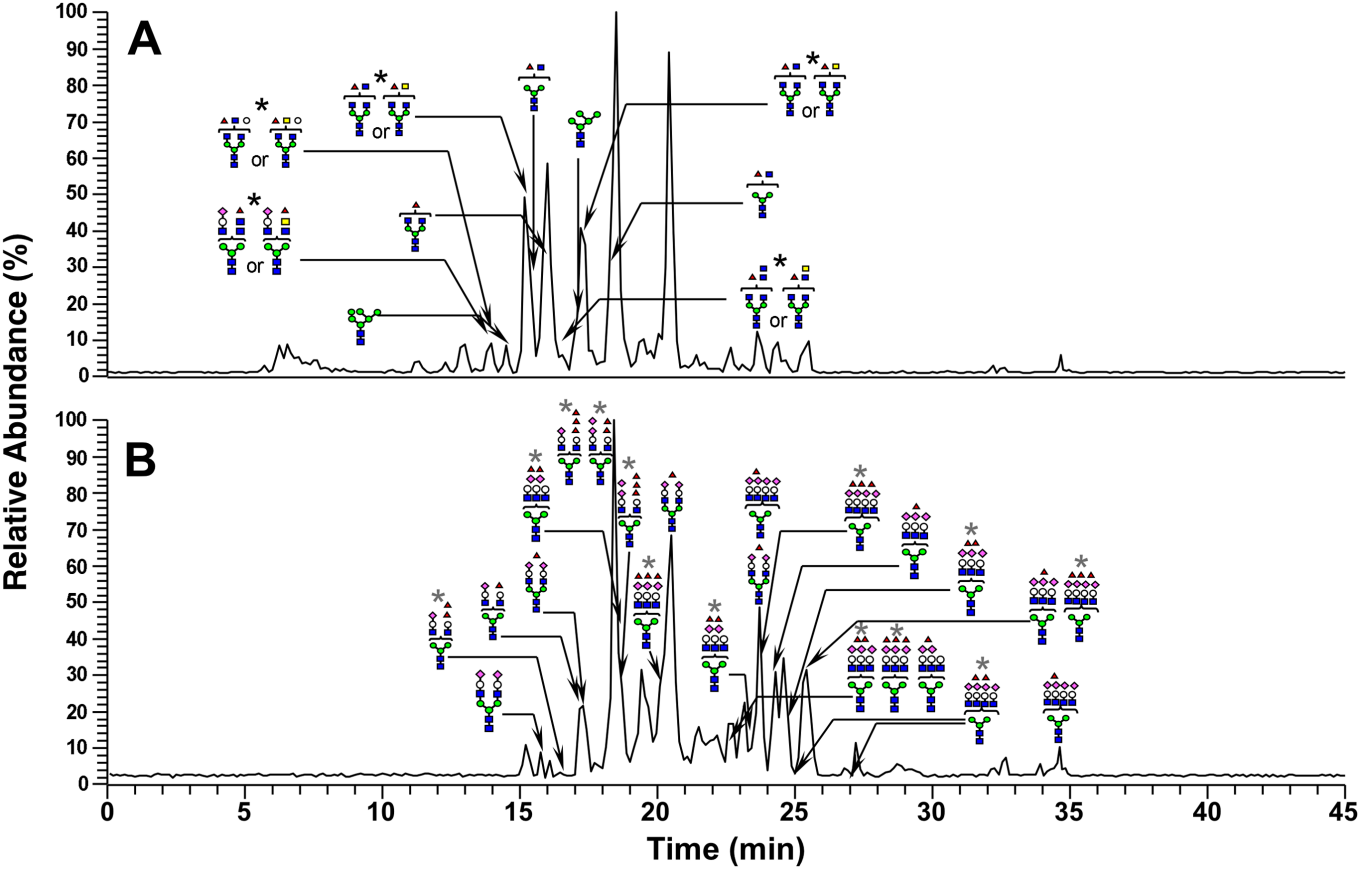
The total ion current chromatogram of *N*-linked glycans from the 70-kDa band obtained by FT-ICR-MS (*m/z* 700–2,000). The positive-ion mode (A) and the negative-ion mode (B). Green circle: mannose, white circle: galactose, red triangle: fucose, blue square: GlcNAc, yellow square: GalNAc, pink diamond: NeuNAc, black asterisk: glycan carrying bisecting GlcNAc or LacdiNAc, gray asterisk: glycan carrying Lewis or sialyl Lewis motif.

The proteins in the 38- and 70-kDa bands were carboxymethylated, followed by digestion with PNGase F. The released *N*-glycans from each band were reduced with sodium borohydride and analyzed by LC/MS. The monosaccharide compositions of the reduced glycans were determined from the accurate masses obtained by FT-ICR-MS. The structures were deduced based on the assignments of fragment ions observed in MS^2^, MS^3^, and MS^4^ spectra. The total ion current chromatogram (TICC) of *N*-glycans from the 38-kDa band obtained by FT-ICR-MS in the positive-ion mode is presented (Fig 3A). Many of the *N*-glycans eluted after 15–20 min were deduced as agalacto-complex-type glycans carrying fucose. As shown in Fig 4A and S1A Fig, the predominant glycan was deoxyhexose (dHex)1 hexose (Hex)3 *N*-acetylhexosamine (HexNAc)5, which can be assigned to a fucosylated agalacto-complex-type biantennary glycan carrying bisecting *N*-acetylglucosamine (GlcNAc) (Fig 4C, (i)) or *N*-acetylgalactosamine (GalNAc)-GlcNAc (LacdiNAc) (Fig 4C, (ii)). Core-fucosylated disialo-biantennary was also detected to a small extent (Fig 5A). In contrast, no glycans were observed in the TICC obtained by FT-ICR-MS in the negative-ion mode, suggesting that highly sialylated glycans were not present in the 38-kDa band (Fig 3B). Similar to the 38-kDa band, the main glycan extracted from the 70-kDa band and detected in the positive-ion mode was a low-molecular-mass glycan carrying bisecting GlcNAc or LacdiNAc (Figs 4B and 5A, S1B Fig). Agalacto- and monogalacto-biantennaries carrying fucose, and high-mannose-type glycans were also detected as major glycans. In addition to these neutral glycans, several acidic glycans were observed at 15–35 min in negative-ion mode (Fig 5B). Interestingly, many of these were highly fucosylated and sialylated complex-type glycans that might possess Lewis or sialyl Lewis motifs (S2 Fig).

### Reactive pattern of lectins against the Ts4-immunoprecipitants

**Fig 6:**
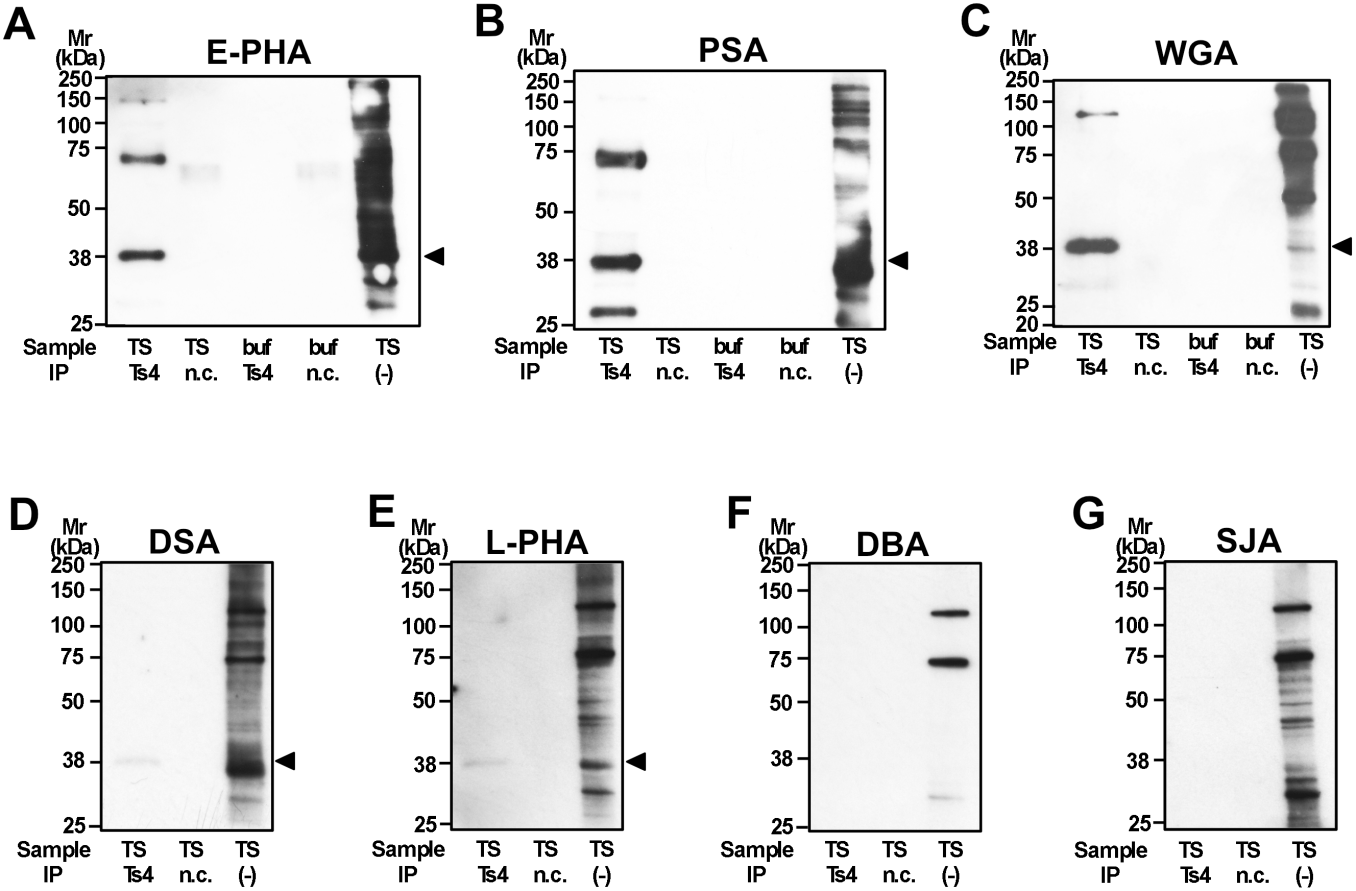
Reactivity of lectins against proteins in the mouse testicular TS fraction co-immunoprecipitated with Ts4. The immunoprecipitated proteins from testicular TS fraction with either Ts4 or normal control IgM (n.c.) were separated by SDS-PAGE under reducing conditions. Control experiments were conducted under the same conditions except for the absence of the TS fraction (buf). The testicular TS fraction was used as a positive control (IP (-)). Proteins were electroblotted onto PVDF membranes and then probed with E-PHA (A), PSA (B), WGA (C), DSA (D), L-PHA (E), DBA (F), or SJA (G). Arrowheads indicate the lectin-reactive bands corresponding to TEX101. Mr, molecular mass.

To determine the *N*-glycan structure that is contained in the Ts4-epitope in the candidates (bisecting GlcNAc or LacdiNAc), we investigated the reactivity of various lectins against the Ts4-immunoprecipitant. Western blot analyses revealed that E-PHA (binding to bisecting GlcNAc [14]), PSA (binding to fucosylated complex-type glycans [15]), and WGA (binding to hybrid-type glycans, bisecting GlcNAc, sialylated complex-type glycans, and agalacto-complex-type biantennary glycans [16, 17]) positively stained the 38-kDa band, corresponding to Ts4-reactive glycoproteins (Fig 6A–6C). The 38-kDa band was faintly detected with either DSA or L-PHA (binding to triantennary complex-type glycans [18, 19]) (Fig 6D and 6E), however, binding activity of DBA and SJA (binding to GalNAc [20, 21]) for the Ts4 immunoprecipitants was not detected (Fig 6F and 6G). These results, combined with experimental data from our LC/MS analyses, indicate that the Ts4-epitope in the 38-kDa band (corresponding to TEX101) contains fucosylated agalacto-biantennary complex-type *N*-glycan with bisecting GlcNAc.

### Reactivity of Ts4 against TEX101 after treatment with endo-/exo-glycosidases

**Fig 7:**
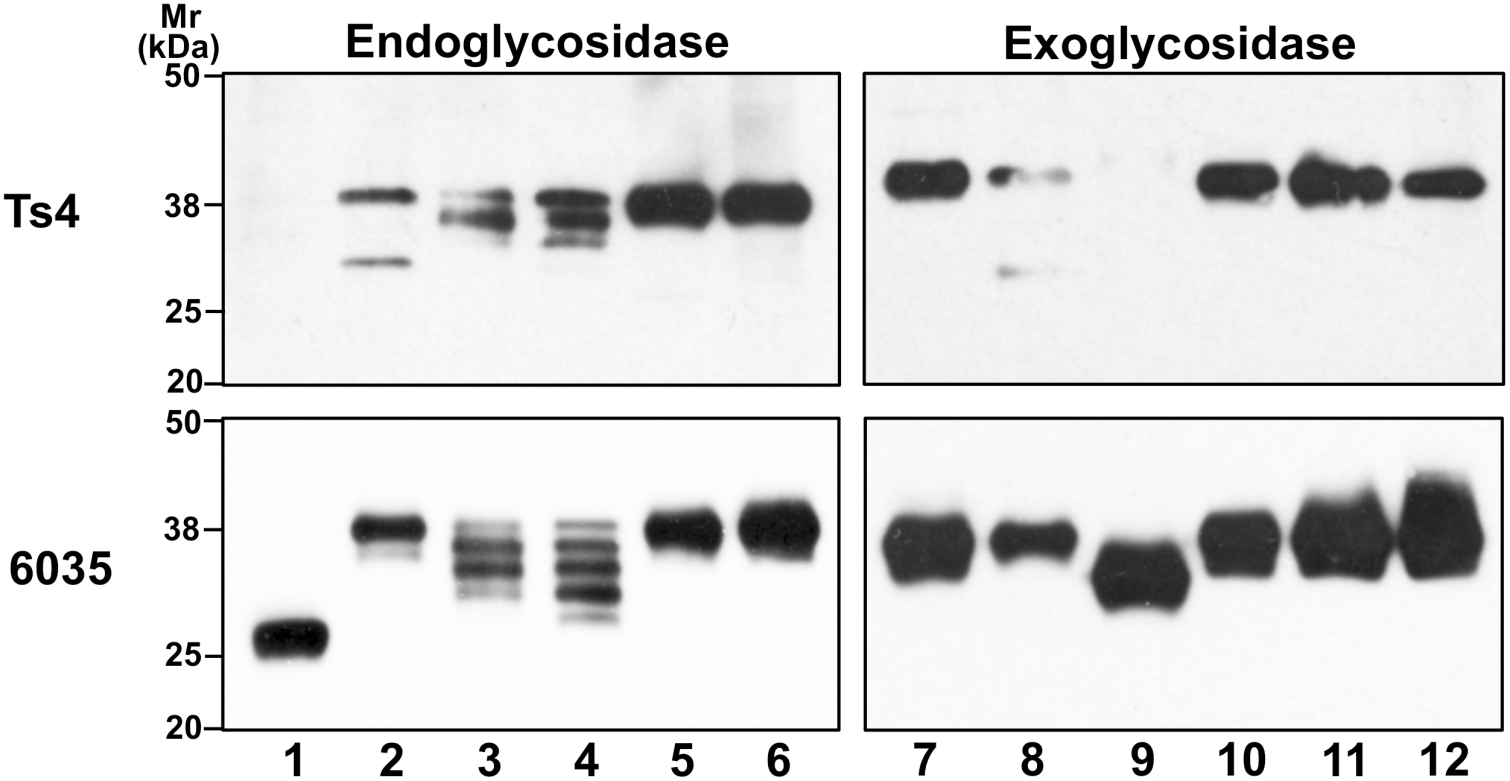
Western blot analysis of the testicular TS fraction after endo- or exo-glycosidase digestion. Aliquots containing 20 μg of testicular TS extract were digested with various kinds of endo- or exoglycosidase. After the reaction mixture was incubated for 24 h at 37°C, 5 μg of proteins was applied to each lane of the 10% SDS-PAGE gel under reducing conditions and then was probed with the Ts4 mAb (upper panel) or the 6035 mAb (lower panel). Lane 1: *N*-glycanase (0.1 unit/ml), lane 2: endoglycosidase F1 (0.1 unit/ml), lane 3: endoglycosidase F2 (0.05 unit/ml), lane 4: endoglycosidase F3 (0.05 unit/ml), lane 5: endoglycosidase H (0.05 unit/ml), lane 6: in absent of endoglycosidase, lane 7: α-mannosidase (7 unit/ml), lane 8: β-mannosidase (0.03 unit/ml), lane 9: β-*N*-acetylglucosaminidase (2 unit/ml), lane 10: neuraminidase (0.16 unit/ml), lane 11: α-L-fucosidase (0.6 unit/ml), lane 12: in absent of exoglycosidase.

To confirm that Ts4 shows immunoreactivity against the epitope containing fucosylated agalacto-biantennary complex-type *N*-glycan with bisecting GlcNAc, we checked pattern of Ts4 reactivity against TEX101 after digestion with various endo-/exo-glycosidases. After treatment with *N*-glycanase, the immunoreactivity of Ts4 against TEX101 was entirely abrogated (Fig 7, lane 1 in upper panel), whereas 6035 recognized an approximately 25-kDa band (Fig 7, lane 1 in lower panel), corresponding to deglycosylated TEX101, as previously described [2]. In similar to the previous studies [2, 4], 6035 detected four or five bands in the testicular TS extract treated with endoglycosidase-F2 (an enzyme that cleaves *N*-linked or free oligomannose and biantennary complex OS chains [22]) or -F3 (an enzyme that cleaves *N*-linked biantennary and triantennary complex OS chains [22]) (Fig 7, lanes 3, 4 in lower panel), respectively. These results indicate that four potential *N*-glycosylation sites within TEX101-peptide sequence [5] were associated with endoglycosidase-F2 and -F3-sensitive OS chains. Among these bands detected with 6035, Ts4 showed immunoreactivity against only three bands at relatively high molecular mass (Fig 7, lanes 3, 4 in upper panel), indicating that Ts4 recognizes only a part of endoglycosidase-F2 and -F3 sensitive OS chains on TEX101 molecules. After endoglycosidase-F1 (an enzyme that cleaves *N*-linked or free oligomannose and hybrid but not complex OS chains [22]) or -H (an enzyme that cleaves *N*-linked high-mannose/hybrid OS chains [22]) digestion, the main Ts4 immunoreaction against the 38-Da band (TEX101) and 6035 mAbs did not change (Fig 7, lanes 2, 5), suggesting that quite a low rate of TEX101 molecules possessed these endoglycosidases-sensitive OS chains. Data obtained from digestive experiments with endoglycosidases strongly suggest that majority of OS chains on TEX101 are *N*-linked biantennary complex type, and Ts4 immunoreacts with only a part of the OS chains.

We next investigated the immunoreactivity of Ts4 and 6035 against TEX101 digested with exoglycosidases. When the testicular TS fraction was treated with α-mannosidase (an enzyme that hydrolyzes terminal non-reducing α-D-mannose residues in α-D-mannosides [23]), neuraminidase (an enzyme that releases sialic acids residues from sialylated glycoconjugates [24]), or α-L-fucosidase (an enzyme that catalyzes the hydrolysis of α-linked L-fucose from the non-reducing end of glycans [25]), the immunoreactivity of both mAbs to TEX101 did not change (Fig 7, lanes 7, 10, 11). However, treatment of the TS extract with β-mannosidase caused partially inhibition of Ts4-reactivity to TEX101 (Fig 7, lane 8 in upper panel). It is known that β-mannosidase cleaves the single β-linked mannose residue from the non-reducing end of *N*-linked OS chains [26]. Thus, these results indicate that the structure of Ts4-recognized OS chain is consistent with the deduced structures proposed as Ts4 epitope (Fig 4C). On the other hand, the immunoreactivity of Ts4 against TEX101 was completely abrogated by pre-treating with β-*N*-acetylglucosaminidase from *Canavalia ensiformis* (Sigma-Aldrich) (Fig 7, lane 9 in upper panel). β-*N*-acetylglucosaminidase is a highly specific exoglycosidase that catalyzes the hydrolysis of terminal non-reducing GlcNAc residues [27]. Among the enzyme sensitive bisecting terminal GlcNAc on the biantennary complex-type *N*-glycans, only a bisected *N*-glycan that possesses the structure shown in Fig 4C, (i) can be cleaved by the enzyme from *Xanthomonas manihotis* (New England Biolabs) (https://www.neb.com/products/p0732-n-acetylglucosaminidase; https://www.neb.com/~/media/NebUs/Files/Application%20Notes/appNoteP0732.pdf). Based on the application note, we further examined the immunoreactivity of Ts4 against TEX101 pre-treated with β-*N*-acetylglucosaminidase from *Xanthomonas manihotis* (New England Biolabs) [27]. As expected, the immunoreactivity of Ts4 against TEX101 was completely abrogated (data not shown), like the enzyme from *Canavalia ensiformis* (Fig 7). Taken together, these results strongly suggest that Ts4 recognizes the epitope containing agalacto-biantennary complex-type *N*-glycan with bisecting GlcNAc.

## Discussion

We previously found that one of the Ts4-reactive testicular proteins (a 38-kDa protein) corresponds to TEX101, a germ cell-specific glycoprotein [2, 5–7], and that the nature of the Ts4-epitope is confirmed as *N*-linked OS chain [2]. Based on the unique distribution of the Ts4-epitope within reproductive-related area [2, 3] (Table 1), we hypothesize that the OS chain would be expected to have a unique structure, and that the glycan has presumably important physiological roles in the fertilization process. In this study, we proposed the chemical structure of *N*-glycan in the Ts4-epitope using an immunoprecipitation method combined with LC/MS.

Although Ts4 detected three major bands at apparent molecular masses of 38, 110, and 140 kDa in the testicular TS extract using Western blot analysis [2] (Fig 1), two major bands at apparent molecular masses of 38 and 70 kDa were observed in the Ts4-immunoprecipitants separated by SDS-PAGE with CBB staining (Fig 2). Amongst the two bands observed, the 70-kDa band was not always shown under denatured conditions (Fig 1, lane 1) [2, 3]. This phenomenon suggests that Ts4 might recognize a conformational epitope, which would retain when pull down by the mAb but be destroyed by glycosidases or other treatments. As supported the hypothesis, the 70-kDa band in the testicular TS fraction was transiently detected by Western blot analyses with Ts4 after periodic acid treatment for 15 min, and immediately disappeared in samples treated for longer incubation times (Fig 1). This observation may be because the conditions used in the periodic acid treatment for testicular TS fractions transblotted on PVDF membranes affect the OS chain(s) of the 70-kDa testicular glycoprotein, like exo- and/or endo-glycosidases. As a result, the OS chains reactive with Ts4 were exposed as terminal residues approximately 15 min after treatment. Further treatment may completely destroy the steric structure of Ts4-reactive OS chains on the 70-kDa glycoprotein. During Ts4-immunoprecipitation of the testicular TS fraction, a similar situation may occur on the sugar residues of the 70-kDa protein. Accordingly, an additional 70-kDa band was observed in the Ts4-immunoprecipitants detected with the same mAb (Fig 2A), and the 70-kDa as well as the 38-kDa bands were detected as major bands in the Ts4-immunoprecipitants stained with CBB (Fig 2B). Therefore, we used these two bands as the target material for further structural analyses of *N*-linked OS chain(s) using LC/MS.

Compared to structural profiles obtained from LC/MS analyses for *N*-linked OS chains from the 38-kDa and 70-kDa bands, the common OS structures observed in both bands are bisecting GlcNAc and LacdiNAc (Figs 3–5 and S1 and S2 Figs). Further binding analyses of the both bands indicate that among the specific sugar-binding molecules examined, clear reactivity was observed only in E-PHA and PSA (Fig 6A and 6B), but not in DBA or SJA (Fig 6F and 6G). Although WGA showed negative binding to the 70-kDa band which is somewhat strange (Fig 6C), this reaction deficiency of the lectin to 70-kDa band may be due to micro-structural changes during long immunoprecipitation time (rotary shaking for overnight at 4°C). This speculation is supported by the experimental result that Ts4 has no reactivity toward the 70-kDa band in the TS fraction before immunoprecipitation [2] (Fig 1, lane 1). Therefore, these results, combined with data from LC/MS analyses, allow us to conclude that fucosylated agalacto-biantennary complex-type *N*-glycan with bisecting GlcNAc is, at least in part, the Ts4-epitope on TEX101 (the 38-kDa band). We additionally observed changes in the immunoreactivity of Ts4 against the 38-kDa band in the TS fraction digested by endoglycosidase-F2, -F3, β-mannosidase, and β-*N*-acetylglucosaminidase, respectively (Fig 7). These results also confirm that the structure of Ts4-recognized molecule contains agalacto-biantennary complex-type *N*-linked OS chain with bisecting GlcNAc.

As a final validation, direct binding between Ts4 and a chemically synthesized OS chain (fucosylated agalacto-biantennary complex-type *N*-glycan with bisecting GlcNAc) should be shown. For this purpose, we preliminary examined direct binding using a glycan array method with several synthesized OS residues. However, we did not obtain any convincing results from the array experiments. In the glycan array method we used, the positive control (lectins showing for specific sugar reactivity, including toward human blood group OS) showed traces of signal, presumably due to sensitivity limitation of the glycan-binding molecules [28]. Our previous report demonstrated that a mAb against hamster oviduct-specific glycoprotein [29, 30] showed immunoreactivity for the OS chain on the protein possessing an identical structure of human blood group A OS [31]. Interestingly, this mAb shows an immunoreactivity for glycolipids isolated from the stroma of human A group red blood cells (RBC) monitored with conventional enzyme immunoassay on a thin-layer chromatography plate, like commercially available specific antibodies against human A group RBC (Araki, unpublished results). These results imply that the glycan-binding molecule (mAb) can react with common OS structures on either glycoprotein or glycolipid. However, poor reactivity of positive controls in glycan array method compared to carbohydrate-complexes (glycoproteins and/or glycolipids) may suggest the necessity of basic structures of OS association sites (*O*-/*N*-glycosylation sites of peptides, or ceramide) for full binding activity of glycan-binding molecules.

It is widely believed that understanding the structure and function of OS chains has significant utility because this class of molecule has been implicated in various physiological and pathological phenomena [8]. Despite their potential significance, structural analyses of OS chains have been studied much less often than other biomolecules, such as peptides and nucleotides, due to the technical limitations of carbohydrate analyses in general. Therefore, the development of reliable methods for carbohydrate structure analyses is indeed essential for further exploration into the world of life science. For this purpose, MS methods have advanced to achieve more routine, reliable, and comprehensive composition analyses in recent years [32]. However, current performance is not always sensitive enough, particularly for analyses of materials originating from mammalian germ cells. Several analytical technologies for OS chains are currently being developed but further improvement is necessary, in particular for mammalian (including human) materials with quantitative collection limitations, such as germ cells.

The bisecting GlcNAc is a unique modification found in various hybrid and complex *N*-glycans, and is an enzymatic product of *N*-acetylglucosaminyltransferase III (GnT-III) that transfers GlcNAc from uridine diphosphate-GlcNAc to core mannose with a β-1,4 linkage [33]. No additional processing of OS chain occurs in *N*-glycans possessing bisecting GlcNAc residue, because other glycosyltransferases (e.g. GnT-IV, GnT-V, and GnT-VI) can not utilize the *N*-glycans with bisecting GlcNAc as a substrate [34]. Therefore, GnT-III and the bisected *N*-glycans are thought to play unique roles in biological functions. For instance, the transfection of *GnT-III* into B16 melanoma cells reduced its metastatic ability via remodeling OS chains of adhesion molecules, such as α_5_β_1_ integrin and E-cadherin [35, 36]. Also, GnT-III is reported to add bisecting GlcNAc to epidermal growth factor receptor (EGFR) on plasma membrane of tumor cells [37]. This modification of OS chain inhibited binding of a ligand to EGFR, resulting that cell differentiation and proliferation were suppressed [38]. Thus the bisecting GlcNAc structures are believed to be involved in biological functions, such as cell adhesion, migration, and growth by regulating on cell surface glycoproteins.

Although biological functions of the bisecting GlcNAc in germ cells are unknown, we recently reported that TEX101 (a bisecting GlcNAc-conjugated glycoprotein)-deficient mice were infertile for inability of spermatozoa to migrate from the uterus into the oviduct and to bind to the zona pellucida [39]. In addition, we have already found that Ts4 has an inhibitory effect on binding of spermatozoa to eggs [40], although Ts4-recognized glycoproteins on the acrosomal region of epididymal spermatozoa are still unknown. These findings lead us to speculate that the bisecting GlcNAc has a potential role in fertilization by modulating biological functions of the glycoproteins related to cell adhesion and migration. We are currently attempting to identify the Ts4-reactive glycoproteins in the epididymal spermatozoa using a proteomic approach.

To date, several unique cancer-specific carbohydrate antigens have been reported, and biosynthesis and functional studies have been conducted [41]. The bisecting GlcNAc is also reported to play important roles in tumor progression and metastasis [42, 43]. Since expression of glycans among early embryos, cancers and stem cells can be correlated [44], the unique *N*-glycan identified in the present study should have common biological functions in reproductive processes and cancer biology. The Ts4 mAb will be a useful tool for further analysis of the bisecting GlcNAc, including its functions.

## Supporting Information

S1 Fig Glycan distributions based on percentage ratios of the peak areas (positive-ion mode) of glycans. The 38-kDa (A) and 70-kDa (B) glycans. Numbers in parentheses represent isomers. Green circle: mannose, white circle: galactose, red triangle: fucose, blue square: GlcNAc, yellow square: GalNAc, pink diamond: NeuNAc, Asterisk: glycan carrying bisecting GlcNAc or LacdiNAc.
(TIF)

S2 Fig Glycan distributions based on percentage ratios of peak areas (negative-ion mode) of glycans from the 70-kDa band. Numbers in parentheses represent isomers. Green circle: mannose, white circle: galactose, red triangle: fucose, blue square: GlcNAc, yellow square: GalNAc, pink diamond: NeuNAc, Asterisk: glycan carrying Lewis or sialyl Lewis motif.
(TIF)
