# Supplementary figures and images for: Chemical Characterization of N-Linked Oligosaccharide As the Antigen Epitope Recognized by an Anti-Sperm Auto-Monoclonal Antibody, Ts4
Source: PLoS One. 2015 Jul 29;10(7):e0133784. doi: 10.1371/journal.pone.0133784 (PMC4519047; doi:10.1371/journal.pone.0133784)

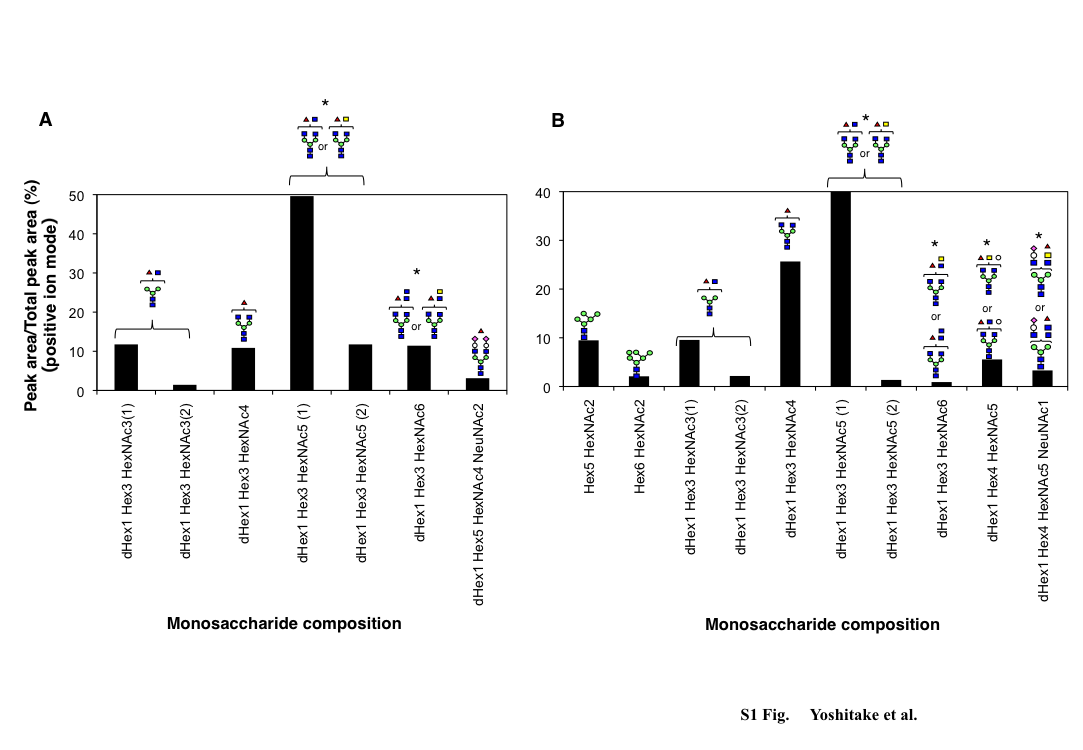

Supplement: S1 Fig — The 38-kDa (A) and 70-kDa (B) glycans. Numbers in parentheses represent isomers. Green circle: mannose, white circle: galactose, red triangle: fucose, blue square: GlcNAc, yellow square: GalNAc, pink diamond: NeuNAc, Asterisk: glycan carrying bisecting GlcNAc or LacdiNAc. (TIF) [file pone.0133784.s001.tif]

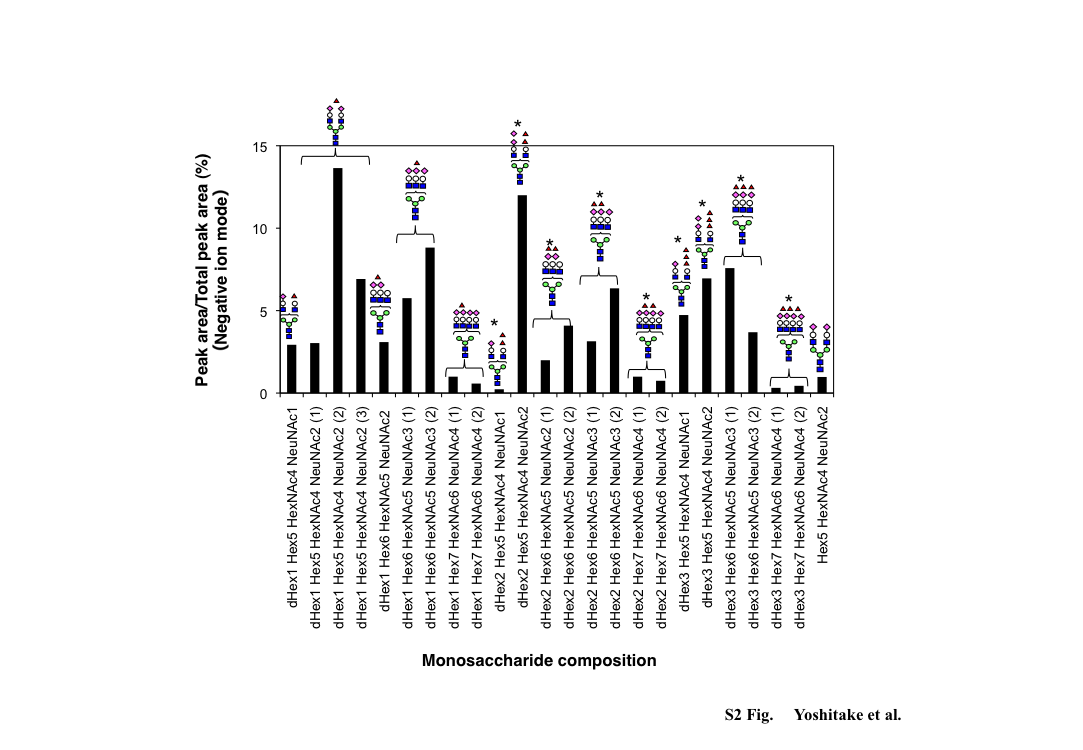

Supplement: S2 Fig — Numbers in parentheses represent isomers. Green circle: mannose, white circle: galactose, red triangle: fucose, blue square: GlcNAc, yellow square: GalNAc, pink diamond: NeuNAc, Asterisk: glycan carrying Lewis or sialyl Lewis motif. (TIF) [file pone.0133784.s002.tif]
